# Non-Isothermal Crystallization Kinetics of PBSu/Biochar Composites Studied by Isoconversional and Model Fitting Methods

**DOI:** 10.3390/polym15071603

**Published:** 2023-03-23

**Authors:** Katerina Papadopoulou, Evangelia Tarani, Konstantinos Chrissafis, Ondřej Mašek, Dimitrios N. Bikiaris

**Affiliations:** 1 Laboratory of Polymer Chemistry and Technology, Department of Chemistry, Aristotle University of Thessaloniki, GR-54124 Thessaloniki, Greece; 2 Laboratory of Advanced Materials and Devices, Department of Physics, Aristotle University of Thessaloniki, GR-54124 Thessaloniki, Greece; 3 UK Biochar Research Centre, School of GeoSciences, University of Edinburgh, Alexander Crum Brown Road, Edinburgh EH9 3FF, UK

**Keywords:** non-isothermal crystallization kinetics, poly(butylene succinate), biochar, Nakamura/Hoffman–Lauritzen model, Šesták–Berggren/Hoffman–Lauritzen model

## Abstract

Non-isothermal crystallization of Poly(butylene succinate) (PBSu)/biochar composites was studied at various constant cooling rates using differential scanning calorimetry. The analysis of the kinetics data revealed that the overall crystallization rate and activation energy of the PBSu polymer were significantly influenced by the addition of biochar. Specifically, the PBSu/5% biochar composite with a higher filler content was more effective as a nucleation agent in the polymer matrix, as indicated by the nucleation activity (ψ) value of 0.45. The activation energy of the PBSu/5% biochar composite was found to be higher than that of the other compositions, while the nucleation activity of the PBSu/biochar composites decreased as the biochar content increased. The Avrami equation, which is commonly used to describe the kinetics of crystallization, was found to be limited in accurately predicting the non-isothermal crystallization behavior of PBSu and PBSu/biochar composites. Although the Nakamura/Hoffman–Lauritzen model performed well overall, it may not have accurately predicted the crystallization rate at the end of the process due to the possibility of secondary crystallization. Finally, the combination of the Šesták–Berggren model with the Hoffman–Lauritzen theory was found to accurately predict the crystallization behavior of the PBSu/biochar composites, indicating a complex crystallization mechanism involving both nucleation and growth. The K_g_ parameter of neat PBSu was found to be 0.7099 K^2^, while the melting temperature and glass transition temperature of neat PBSu were found to be 114.91 °C and 35 °C, respectively, very close to the measured values. The Avrami nucleation dimension n was found to 2.65 for PBSu/5% biochar composite indicating that the crystallization process is complex in the composites.

## 1. Introduction

Thermal analysis techniques are useful for studying the crystallization kinetics of polymers. These techniques can be used to investigate how the crystallization rate is influenced by various factors, such as temperature, cooling rate, and the presence of additives, and to understand the underlying mechanisms of the crystallization process [1]. However, it is important to carefully consider the phenomena occurring during crystallization, the temperature program used in the experiment (whether it is isothermal or dynamic), and the nature of the material being studied before choosing an appropriate model and determining the rate constants for the process. Using an incorrect model or rate constant can lead to results that are physically meaningless or misleading. Non-isothermal crystallization kinetics makes it easier to study the processes and factors that affect the rate of crystallization when the temperature changes. Non-isothermal kinetics can be used to determine the kinetic triplet, which is made up of the activation energy, pre-exponential factor, and reaction model. These characteristics can be measured using various scanning speeds in differential scanning calorimetry (DSC) and then utilized to forecast non-isothermal crystallization kinetics according to ICTAC standards [2,3].

Biomass-derived biobased polymers and additives can be produced in large quantities from plants through photosynthesis using CO_2_ and water. Poly(butylene succinate) (PBSu) is a biobased polymer produced by a two-stage melt polycondensation process from biobased succinic acid and butanediol [4,5]. PBSu is a semi-crystalline polyester with similar characteristics to fossil-derived polymers, e.g., thermomechanical properties and good chemical resistance, such as polypropylene (PP) and low-density polyethylene (LDPE) [6]. It is biodegradable under certain conditions and can be recycled for use in applications such as food packaging, single-use items, agriculture, and automotive as alternatives to traditional non-degradable polymers. To enhance the properties of the polyester, a biobased additive known as biochar was used as a reinforcing agent. Biochar is a solid substance made by heating biomass in an oxygen-deprived environment at high temperatures (300–800 °C) The structure of biochar and its properties are determined by a plethora of factors, including pyrolysis conditions, particle size and type of feedstock [7]. It is being explored as a cost-effective and environmentally friendly alternative [8,9] to traditional carbon fillers in the creation of polymer-based composites. Biochar has high thermal stability, a large surface area, and is electrically conductive, and its properties can be adjusted through the selection of starting biomass and production process conditions [10]. It is being considered as a potential replacement for other high-performing fillers in the creation of multi-functional polymer-based composites due to its ability to improve mechanical, electrical, and thermal properties. Additionally, biochar can be made from a wide range of biomass sources, including agricultural waste, which makes it a potential contributor to a circular bioeconomy that aims for a circular bioeconomy [11].

PBSu/biochar composites have gained increasing attention in recent years due to their potential use in various applications. These composites have shown promise as adsorbents for removing pollutants from water and as catalysts for producing biofuels [12,13,14]. On the one hand, one of the key properties of PBSu/biochar composites is their ability to adsorb various types of pollutants from water [14]. This property is largely due to the high surface area and porous structure of the biochar, which allow it to effectively capture and retain contaminants. On the other hand, PBSu adds strength and stability to the composite, helping to prevent the biochar from breaking down over time. In addition to their use as adsorbents, PBSu biochar composites have also been explored as catalysts for the production of biofuels [12,13]. In this application, the biochar serves as a support material for the catalyst, while the PBSu helps to improve the catalytic activity and stability of the composite. One of the main challenges in the use of PBSu biochar composites is the optimization of their properties and performance This can be achieved through the careful selection of the biochar and PBSu materials and the processing conditions used to produce the composite. From our previous work it was found that the fine dispersion of biochar in PBSu matrix improves the tensile and impact strengths [15]. Further research and development in this area could lead to the creation of new and improved PBSu biochar composites with enhanced capabilities and wider applications.

The Johnson–Mehl–Avrami–Erofeev–Kolmogorov (JMAEK) equation is often used to fit experimental data of PBSu composites because it allows for the determination of the rate constant and Avrami exponent, which can provide information about the mechanism and kinetics of non-isothermal crystallization. Qiu et al. [16] used a modified version of the JMAEK equation to study the non-isothermal melt crystallization of PBSu at different cooling rates, ranging from 1 to 10 °C/min. The results showed that the Avrami exponent (n) and the logarithm of the reaction rate constant (logk) values varied significantly with the cooling rate, making it difficult to accurately understand the overall crystallization process. The resulting Avrami exponent values ranged from 4.1 to 5.7. Additionally, Gao et al. [17] showed that the n values for PBSu/magnesium hydroxide sulfate whisker composites fall within a range of 5.27–6.69, which are higher than those with a physical meaning. Bin et al. [18] studied the non-isothermal crystallization kinetics of PBSu using the Avrami model, the Ozawa model, and the Liu model (modified Avrami–Ozawa model). They found that the Ozawa model was unable to accurately describe the crystallization behavior of PBSu, while the Avrami model and the Liu model were able to provide satisfactory descriptions. However, they also noted that it is difficult to assign a physical meaning to the parameters Z_t_ and n related to the non-isothermal crystallization of PBSu [19]. Although there have been several studies on the crystallization process of neat PBSu, there has not yet been a comprehensive analysis of the melt crystallization and crystallization kinetics of PBSu/biochar composites. This lack of research raises concerns about the usefulness of the above-mentioned methods for analyzing the activation energy, rate constant, and Avrami exponent of these materials under linear heating conditions.

This study examined the effect of various amounts of biochar on the crystallization and melting of PBSu. Biochar was used to make composites at concentrations of 1%, 2.5%, and 5 wt.% in the PBSu matrix using an in situ polymerization method. To quantify the effect of the biochar filler on the PBSu matrix, the nucleation activity parameter (ψ) and the half time of crystallization values (t_1/2_) were calculated. Crystallization kinetics of PBSu biochar composites in non-isothermal conditions was studied at several constant cooling rates, from 1 to 10 °C/min, by means of DSC. Both isoconversional and model-based approaches were used to determine the kinetic triplet, including the activation energy, preexponential factor, and reaction model. The Friedman and Vyazovkin analyses were used to calculate crystallization energy using isoconversional methods, while the Avrami–Erofeev equation (An), the Nakamura/Hoffman–Lauritzen model, and the Šesták–Berggren model (SB) with the Hoffman–Lauritzen theory were employed for model-based analysis. This is the first time that simulation equations for the crystallization process of PBSu/biochar composites using kinetic analysis have been presented; it compares the performance and effectiveness of these methods in analyzing crystallization processes.

## 2. Materials and Methods

For the preparation of PBSu/biochar composites, the following reagents were used: succinic acid (SA) (purum 99+%), and titanium isopropoxide (≥97%) (Tis) catalyst of analytical grade, which were purchased from Sigma-Aldrich Chemical Co (Saint Louis, MO, USA), and 1,4-Butanediol (BD) (Purity: >99%), which was obtained from Alfa Aesar (Kandel, Germany). Biochar was synthesized from Miscanthus Straw Pellets using the Stage III pilot-scale pyrolysis unit at 700 °C [13]. It was provided by UK Biochar Research Centre. Before use, the biochar was dried overnight in an oven at 80 °C under vacuum. All other reagents were of analytical grade.

The method of two stage polycondensation reaction (esterification and polycondensation) took place. Composites of PBSu and biochar were prepared using SA and BD in a molar ratio 1/1.1 and biochar at concentration of 1%, 2.5% and 5 wt.%, which was added in the glass batch reactor simultaneously with SA and BD [20,21]. At the beginning of the reaction, the reaction mixture was heated to 170 °C under nitrogen flow for 1 h and stirring speed 500 rpm, subsequently at 180 °C for additional 1 h, and finally at 190 °C for 1.5 h. The first step of esterification was considered complete after the collection of the distilled amount of water in a graduated cylinder. In the stage of polycondensation (second step), the vacuum was gradually increased to 5.0 Pa over a period of about 30 min, to remove the excess diol or remaining H_2_O, to avoid excessive foaming and to minimize oligomer sublimation. During this period, the temperature was gradually increased to 230 °C, while stirring speed was also increased to 720 rpm. A total of 400 ppm of Tis catalyst was added in the reactor at the end of this stage. At this temperature, the reaction was kept constant for 30 min, and every 30 min was increased 10 °C until reaching 250 °C, where the reaction was continued for 1.5 h (total polycondensation time, 2.5 h). As the polycondensation reaction was completed, the polyesters were easily removed from the flask.

The resulting samples’ FTIR (Fourier-transformed infrared spectroscopy) spectra were acquired using the FTIR-2000 (Perkin Elmer, Waltham, MA, USA). Compression molding at 180 °C in a thermopress was used to create thin films (less than 100 μm). All spectra were gathered at a resolution of 4 cm^−1^ and 32 co-added scans in the range of 4000 to 450 cm^−1^.Using an FEI Tecnai G2 20 microscope (FEI, Hillsboro, OR, USA) and an accelerating voltage of 200 kV, Transmission Electron Microscopy (TEM) examinations were carried out on the samples. To prepare the samples, thin films of neat PBSu and its biocomposites were cut with an ultra-microtome (a DiATOME 45 diamond knife, DiATOME Ltd., Nidau, Switzerland) to a thickness of 80 nm. The thin parts that were resting on the knife’s water surface were placed on grids coated with carbon before being air-dried for the night. The crystallization and melting behavior of PBSu/biochar composites was investigated using a Polyma 214 DSC instrument from NETZSCH, which was calibrated using Indium and Zinc standards. The samples, each weighing approximately 6.5 ± 0.2 mg, were sealed in aluminum pans and analyzed using the DSC. In the non-isothermal crystallization analysis, the thermal history of all the samples was erased by heating from 0 °C to 200 °C at a rate of 20 °C/min under a nitrogen flow of 40 mL/min. The samples were maintained at a specific temperature for 3 min. For the non-isothermal crystallization, cooling scans were performed at rates of 1, 2.5, 5, and 10 °C/min. The non-isothermal crystallization kinetics of the samples were analyzed using the NETZSCH Kinetics Neo software (NETZSCH, Selb, Germany) [22]. DSC curves can be used to calculate the crystallinity fraction of a sample using the following equation:

(1) $$X_{c}=\frac{{\Delta H}_{m}}{\left(1-w\right)\cdot {\Delta H}_{m}^{0}}\cdot 100\%$$

where ΔH_m_ and ${\Delta H}_{m}^{0}$ represent the measured heat of fusion and the heat of fusion of a fully crystalline material, respectively, and w is the weight fraction of the filler that has been incorporated into the polymer matrix.

## 3. Results and Discussion

### 3.1. Structural and Morphological Characterization of PBSu/Biochar Composites

**Figure 1:**
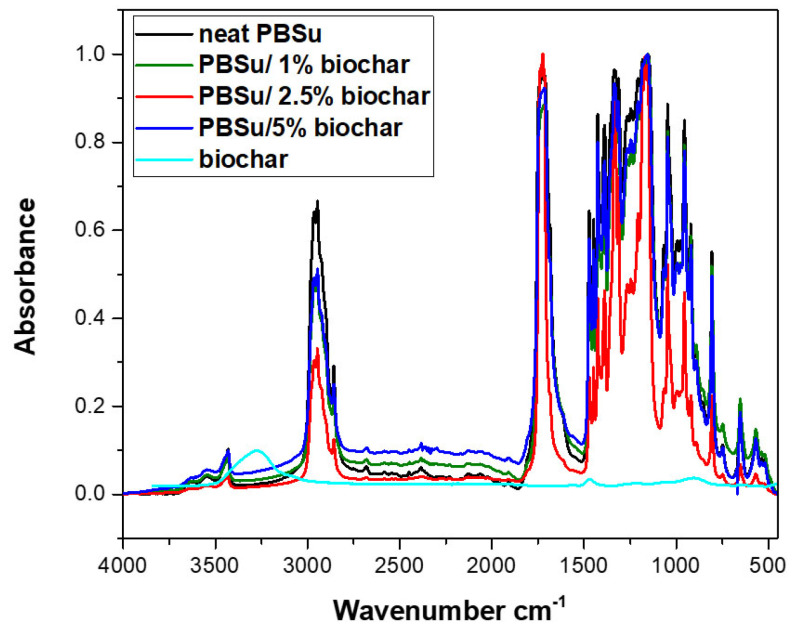
FT-IR spectra of neat PBSu and PBSu–biochar biocomposites.

The successful synthesis of the studied aliphatic polyester and the biochar was also confirmed by FTIR spectroscopy. In Figure 1, the spectra of all samples certainly share similarities because of the alike chemical structure deriving from succinic acid and the 1,4-butanodiol.

The spectrum of neat PBSu in Figure 1 has a broad peak in the range of 3000–2800 and 1600 cm^−1^, which is related to the stretching and bending vibration of the C–H bonds. The characteristic ester absorption peak for the stretching vibration of the >C=O bond appears at 1716 cm^−1^ while the wide peak at 3400–3700 cm^−1^ corresponds to hydroxyl groups of the polyester. From the spectrum of the biochar, it can be seen that the peaks are negligible due to the absence or limited presence of functional groups on surface and high percentages of carbon content. For the PBSu–biochar biocomposites, the IR spectra retained most of the band of PBSu, although the intensity of some of these bands changed. No shifts in characteristic peaks are in accordance with the study of Hernandez-Charpak, et al. [23]. Thus, it seems that no interactions between biochar and PBSu matrix took place. However, there is also the possibility due to the low amount of added biochar (1, 2.5, 5%), these interactions could not be detected with FTIR, due to its low sensitivity.

**Figure 2:**
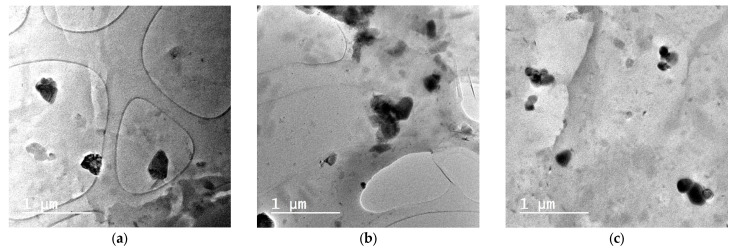
TEM micrographs of PBSu/biochar composites filled with (**a**) 1 wt.% biochar, (**b**) 2.5 wt.% biochar and (**c**) 5 wt.% biochar.

The most important properties of nanocomposites are mainly their mechanical and thermal properties, which depend on the dispersion of nanoadditives and the evolved interface interactions of the nanoadditive with the polymer matrix. In the present study, the dispersion of biochar into the PBSu matrix was studied by TEM. As can be seen from Figure 2, there is a fine dispersion of biochar in all nanocomposites. Black and almost spherical particles are detected with sizes in the nanosized level. Furthermore, it is clear that some aggregates are formed in the nanocomposites containing 2.5 and 5 wt.% biochar, but even in these samples the sizes of aggregates are not larger than 1 μm. From these micrographs, we can conclude that the in situ technique that was used for the preparation of nanocomposites is appropriate to break down the initial large particles of biochar (10–15 μm) at nanosized level [15].

### 3.2. Non-Isothermal Melting and Crystallization Behavior of PBSu/Biochar Composites

**Figure 3:**
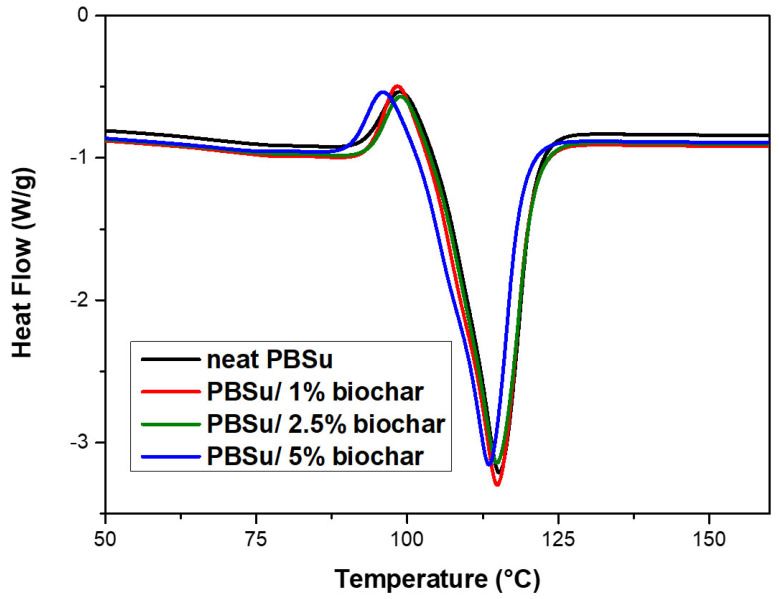
Melting peak temperatures of neat PBSu and PBSu/biochar composites at the heating rate of 20 °C/min.

The impact of biochar on the melting and crystallization behavior of PBSu was studied using DSC. Crystallization is a complex process that involves at least two different steps known as nucleation and growth. Non-isothermal crystallization is classified into two types: crystallization that occurs when the temperature rises from below the glass transition temperature (cold crystallization), and crystallization that occurs when the temperature falls from above the melting temperature (melting crystallization). Figure 3 shows the melting curves of neat PBSu and PBSu/biochar composites filled with various filler contents. An overlap of the boundaries of the cold crystallization peak with the melting peak was found for all the studied samples. The melting curve has a cold crystallization peak, which indicates that the material crystallizes upon cooling from the melt. The overlap of the melting peak and the cold crystallization peak in the DSC measurements suggests that crystallization is occurring during the melting process. Generally, the cooling rate has a significant impact on the crystallization behavior of PBSu, which is affected by the formation of imperfect crystals at low temperatures. Arandia et al. [24] showed that PBSu exhibits cold crystallization during the scan followed by a double melting peak. The presence of two melting peaks could be due to a partial melting and recrystallization process during the heating scan or to the melting of two populations with different mean lamellar thickness. Additionally, the cold crystallization of PBSu was reported by Klonos et al. [25] and was most likely caused by incomplete crystallization during cooling and/or continued evolution of nucleation before melting. Table 1 lists the peak melting temperature (T_m_), the cold crystallization temperature (T_cc_), the fusion enthalpy (ΔH_m_), and the corresponding crystallinity for all the materials studied in this work. Data were collected at a heating rate of 20 °C/min. Table 1 shows how the melting and cold-crystallization temperatures and the fusion enthalpy are affected by the amount of biochar in the PBSu matrix. These results suggest that the addition of biochar can alter the melting and crystallization behavior of PBSu. In detail, the crystallinity of neat PBSu was found to be 33.4%, consistent with previous literature results [26]. As the filler content increases, the melting enthalpy and crystallinity of PBSu/biochar composites both increase, while the melting temperature decreases. This means that the specific effects of the biochar depend on how much biochar is in the composite. Thus, the addition of 5 wt.% of biochar filler shifts the T_m_ to considerably lower temperatures compared to those of neat PBSu and PBSu/biochar composites filled with 1 wt.% and 2.5 wt.%, suggesting the formation of less thermally stable crystals. Thus, the presence of fillers in a polymer can significantly impact the crystallization process and the resulting properties of the material; the fillers may act as nucleation sites that promote the growth of crystals, enhancing crystallinity [27]. The incorporation of biochar was also found to shift the cold crystallization to lower temperatures as the filler content increased. This change means that the biochar has a nucleating effect on the PBSu crystallization, which means that it encourages the formation of crystal nuclei and speeds up the crystallization process. Bosq et al. [28] showed that the crystallization temperatures of PBSu/graphene nanosheets were higher when cooling from a melted state and lower when heating from a glassy state.

**Figure 4:**
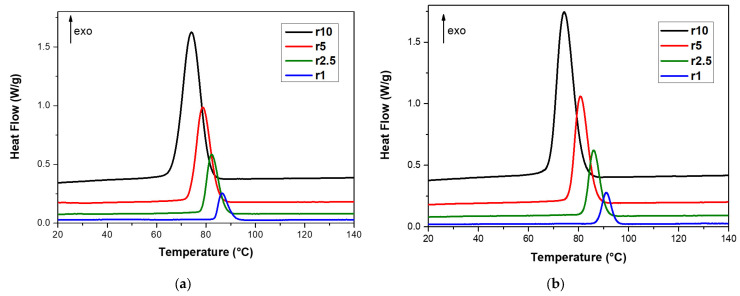
DSC cooling curves of (**a**) neat PBSu and (**b**) PBSu/5% biochar at cooling rates from 1 to 10 °C/min.

To find out how different amounts of biochar affect the crystallization of PBSu/biochar composites, the material was cooled at different rates, from 1 to 10 °C/min. The cooling curves of some selected samples, neat PBSu and PBSu/5% biochar, are shown in Figure 4. All curves show one well-defined exothermal peak. The lowest cooling rates, 1 and 2.5 °C/min, produce a narrow crystallization peak, while the highest cooling rates, 5 and 10 °C/min, produce a broad crystallization curve. The results demonstrated that these materials’ crystallization is significantly influenced by the cooling rate. The specific mechanisms behind this effect depend on the material and the cooling rate used. At higher cooling rates, crystallization of the PBSu/biochar composites occurs at lower temperatures, indicating that the cooling rate can influence the rate of crystallization in these systems. As a result, it is possible to tune the crystallization behavior of these materials by adjusting the cooling rate. The cooling rate’s impact on the material’s undercooling is most likely the cause of the observed shift in crystallization peak temperature with cooling rate [1,29]. The shift to lower temperatures also suggests that the crystallization process becomes more efficient at higher cooling rates, with the molecular chains having less flexibility and taking less time to arrange into more ideal crystallites. Table 2 shows the crystallization temperature T_c_ and enthalpy ∆H_c_ values of PBSu/biochar composites under non-isothermal crystallization conditions. It was found that the crystallization temperature increases with increasing biochar content, at all of the cooling rates tested, between 1 and 10 °C/min. This suggests that the presence of biochar promotes the formation of crystal nuclei and speeds up the crystallization process. Previous research has shown that adding biochar to PP can increase the crystallinity of the resulting composite due to the nucleating effect of the biochar particles [30]. Lee et al. [31] analyzed the non-isothermal crystallization of PBSu/orotic acid composites and found that the crystallization peak temperature of PBSu shifts to higher temperatures when orotic acid is added. Additionally, the addition of biochar to PBSu has been shown to increase the crystallization temperature due to the increased number of nucleation sites provided by the biochar. This was observed in a study by Elnour et al. [32], where the crystallization temperature of a composite material increased with an increase in biochar content.

### 3.3. Nucleation Activity

As mentioned before, the presence of the fillers can alter the crystallization process by promoting the formation of crystal nuclei and thus accelerating the crystallization rate. To quantify the effect of the fillers on the nucleation of the polymer matrix, researchers often use the nucleation activity parameter (ψ), which is a measure of the nucleating ability of the fillers. The nucleation activity parameter can be calculated using non-isothermal crystallization data, and various methods have been proposed for estimating this parameter, including the method suggested by Dobreva et al. [33]:

(2) $$\left(\psi \right)=\frac{B^{*}}{B}$$

where B* and B are the nucleation activity of composite and neat matrix, respectively, which can be experimentally calculated by plotting lnβ versus the inverse squared degree of supercooling 1/ΔΤ^2^:

(3) $$\log\;\beta =\;\mathrm{Const}-\frac{B}{{\Delta Τ}^{2}}$$

where β is the heating/cooling rate and ΔΤ = Τ_m_ − T_mc_ is the melt crystallization data. The model suggested by Dobreva et al. [33] for estimating the nucleation activity parameter (ψ) of fillers in polymer composites is based on the assumption that the fillers have a certain nucleation activity that can influence the crystallization process of the polymer matrix. According to this model, when the nucleation activity parameter (ψ) is close to 0, the filler has a very high nucleation activity and is able to significantly accelerate the crystallization of the polymer. On the other hand, when the nucleation activity parameter is close to 1, the filler has a weaker nucleation activity and has a smaller impact on the crystallization of the polymer.

**Figure 5:**
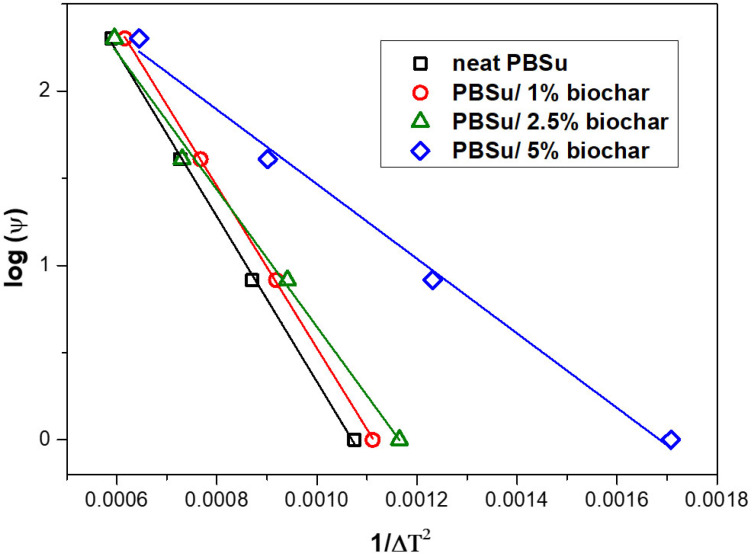
Plots of log ψ versus 1/ΔΤ^2^ for neat PBSu and PBSu/biochar composites filled with various filler content.

Figure 5 shows plots of logβ versus 1/ΔT^2^ for neat PBSu and PBSu/biochar composites filled with various filler contents. From the slopes of these lines, the value B*/B for the neat PBSu, PBSu/1% biochar, PBSu/2.5% biochar and PBSu/5% biochar was calculated to be 1, 0.98, 0.83, and 0.45, respectively. The PBSu/5% biochar composite with a higher filler content was more effective as a nucleation agent in the polymer matrix, as indicated by the nucleation activity (ψ) value. This suggests that the amount of filler in these materials has a big effect on their ability to start new crystals. In detail, when the filler’s content is high, the B*/B ratio takes lower values, suggesting that incorporating larger amounts facilitates heterogeneous nucleation since the available nucleating surface is higher.

The data in Table 1 and Table 2 are consistent with the results shown in Figure 5, which suggest that the nucleation activity (ψ) of the PBSu/biochar composites increases as the filler content increases. Bosq et al. [34] showed that the PBSu/nanoprecipitated calcium carbonate composites exhibited higher nucleation activity than the neat PBSu; the nanoparticles seem to be effective at promoting heterogeneous nucleation, and the presence of filler reduces the amount of supercooling required for nucleation to occur. These results back up the idea that the amount of biochar in the composite changes how the substance crystallizes and that the effects of the biochar are proportional to its amount.

### 3.4. Non-Isothermal Crystallization Kinetics of PBSu/Biochar Composites

The study of processes that are sped up by heat has become very important in the field of material science. Thermal analysis is needed because many of these processes have a direct effect on the quality of the materials that are made in the end. Understanding non-isothermal crystallization kinetics from a practical point of view will be important because most processes for making polymers do not take place in an isothermal setting. In this study, both isoconversional and model-based methods were used to figure out the activation energy, preexponential factor, and reaction model, which together are called the kinetic triplet. The kinetic parameters of the process of melt crystallization were figured out with the help of the NETZSCH Kinetics Neo software. An n-dimensional nucleation model based on the Avrami–Erofeev equation (An), the Nakamura crystallization (Nk) model, and the Šesták–Berggren model (SB) was used for the model-based analysis. The isoconventional approaches included Friedman and Vyazovkin analysis.

Crystallization is a first-order transition of a material, where the crystalline phase is formed from the amorphous state. The crystallization heat of these materials was obtained by measuring the area under the exothermic peak during the crystallization process. The relative degree of crystallinity (X_T_) can be expressed by the following equation:

(4) $$\alpha =X_{T}=\frac{\int_{{Τ}_{0}}^{{Τ}_{c}}\left(\frac{\mathrm{dH}}{\mathrm{dT}}\right)\mathrm{dT}}{\int_{T_{0}}^{T_{\infty }}\left(\frac{\mathrm{dH}}{\mathrm{dT}}\right)\mathrm{dT}}=\frac{{\Delta H}_{T}}{{\Delta H}_{0}}$$

where $T_{0}$, T and $T_{\infty }$ stand for the initial crystallization temperature, the crystallization temperature at time t and the ultimate crystallization temperature, respectively. The enthalpy of crystallization, ΔH, refers to the amount of heat released during the process of a material transitioning from a liquid or amorphous state to a solid, crystalline state. This heat is released over a small range of temperatures. The total heat produced during the entire crystallization process is represented by ΔH_0_. The relationship between crystallization temperature and time can be calculated using the equation:

(5) $$t=\frac{T_{0}-T}{\beta }$$

where $T_{0}$ is the initial crystallization temperature, T is the crystallization temperature at a specific time, and β is the cooling rate.

**Figure 6:**
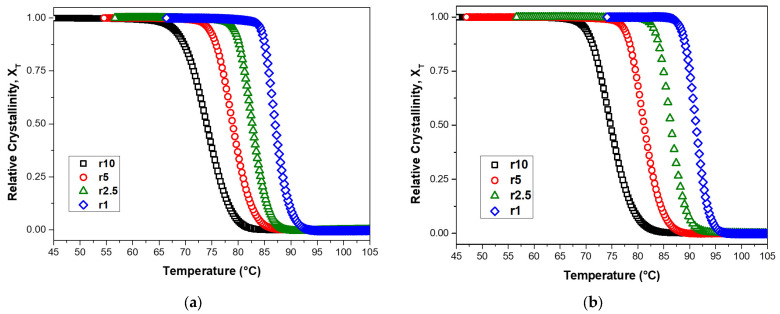
Plots of relative degree of crystallinity as a function of temperature for non-isothermal crystallization of (**a**) neat PBSu and (**b**) PBSu/5% biochar composite at various cooling rates ranging from 1 to 10 °C/min.

Figure 6 shows the relative degree of crystallinity as a function of temperature for non-isothermal crystallization measurements of neat PBSu and PBSu/5% biochar composite. The sigmoidal shape of the curves in Figure 6 suggests that both nucleation and growth are occurring during the crystallization process. The melt crystallization is known to follow anti-Arrhenius behavior, because the crystallization rate decreases with the temperature increase. Even though polymer crystallization is so complicated, it has been described in many ways using single-step models and the Arrhenius law, such as the Avrami equation. Thus, in the early stages of crystallization, nucleation is often the rate-determining step, while diffusion is often the rate-determining step in the later stages.

The figures also demonstrate the impact of the cooling rate on crystallization. The cooling rate, or the rate at which it is cooled from a high temperature, can affect its relative degree of crystallinity. When a material is cooled slowly, it experiences greater undercooling, which is the difference between the melting temperature of the material and the temperature at which crystallization occurs. This allows more time for crystallization to occur, resulting in a higher relative degree of crystallinity. However, as the material cools more quickly, the undercooling diminishes and the crystallization rate increases. In the final stages of crystallization, the curvature of the plot levels off due to the impingement and crowding of spherulites, which are small, spherical structures formed during the crystallization of some polymers. Thus, the presence of spherulites can inhibit further crystallization and lead to the leveling off of the curve.

Table 3 shows how the half-time of crystallization is affected by the amount of filler in neat PBSu and PBSu/biochar composites. The half time of crystallization is defined as the time it takes for the material to reach 50% of the relative degree of crystallinity. These values provide insight into the speed at which the crystallization process occurs for different materials and under different conditions. At a given cooling rate, the half time of crystallization values of PBSu/biochar composites present lower values than those of neat PBSu. The incorporation of biochar into PBSu composites leads to a shorter time for crystallization to reach half completion, indicating a faster crystallization rate. This may be because the presence of biochar provides a site for heterogeneous nucleation, allowing crystallization to start at higher temperatures. The addition of 5 wt.% of biochar to PBSu appears to increase the number of heterogeneous crystallizations. This leads to an overall increase in the crystallization rate. The t_1/2_ values for mica/PBSu composites were less than those for neat PBSu at a fixed cooling rate, according to Zhang et al. [35], indicating that the presence of mica particles speeds up the non-isothermal crystallization process. Yacini et al. [36] found a similar decreasing phenomenon during the non-isothermal process of the PBSu/multi-walled carbon nanotube composites. At a fixed cooling rate, the t_1/2_ values for PBSu/magnesium hydroxide sulfate whisker composites are shorter, indicating that the inclusion of whiskers accelerates the crystallization process and increases the crystallization rate [17]. The decrease in t_1/2_ as the filler content increases is consistent with the results of nucleation activity (ψ) calculations, which also show a decrease in nucleation activity as the filler content increases in PBSu/biochar composites.

#### 3.4.1. Isoconversional Methods of Friedman and Vyazovkin for PBSu/Biochar Composites

The activation energy, E_α_, is a measure of the energy required to initiate and sustain the crystallization process. It is an important parameter in understanding the kinetics of crystallization. There are several methods commonly used to study the crystallization of materials, each with their own advantages and limitations, including the differential isoconversional method developed by Friedman [37], the integral isoconversional methods by Vyazovkin [38] and Ozawa, Flynn, and Wall (OFW) [39]. The activation energy of PBSu/biochar composites was examined in this work using the differential isoconversional method of Friedman and the integral isoconversional method of Vyazovkin. These methods allow for the examination of factors that affect crystallization and the relationship between crystallization temperature and time. The differential isoconversional method of Friedman can be described as follows:

(6) $$\ln\left[\beta_{i}{\left(\frac{\mathrm{da}}{\mathrm{dt}}\right)}_{a,i}\right]=\ln\left[f\left(a\right)A_{a}\right]-\frac{E_{a}}{{\mathrm{RT}}_{a,i}}$$

where A is the pre-exponential factor and β is the heating rate. It is necessary to determine the slope of the straight lines in the plot of ln[β_i_(d/dt)_ai_] vs. 1/T_a,i_ in order to derive the activation energy E values for a constant conversion function. One advantage of the differential method is that it does not require any approximations and can be used with any temperature program. This makes it a flexible and powerful tool for studying the kinetics of chemical reactions and phase transitions. One potential issue with this approach is that it is limited by the precision of the baseline measurement, which may affect the accuracy of the results.

An isoconversional nonlinear method has been proposed by Vyazovkin in order to calculate the E_α_:

(7) $$\Phi \left({Ε}_{\alpha }\right)=\sum_{i=1}^{n}\sum_{j\neq 1}^{n}\frac{J\left[E_{\alpha },{Τ}_{i}\left(t_{\alpha }\right)\right]}{J\left[E_{\alpha },{Τ}_{j}\left(t_{\alpha }\right)\right]}\;$$

where n is the total number of experiments, i and j are a set of experiments performed at different rates of heating, and J is measured during short changes in E_α_ variation:

(8) $$J\left[E_{\alpha },{Τ}_{i}\left(t_{\alpha }\right)\right]=\int_{t_{\alpha -\Delta \alpha }}^{t_{\alpha }}\exp\left[\frac{-E_{\alpha }}{{\mathrm{RT}}_{i}\left(t\right)}\right]\mathrm{dt}$$

**Figure 7:**
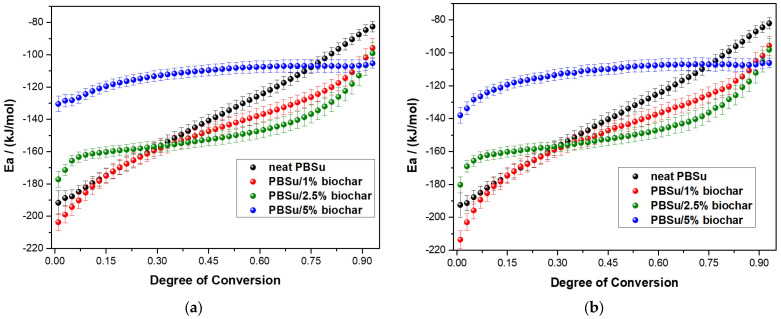
The dependence of activation energy (E_α_) on the degree of conversion (α) for the non-isothermal crystallization kinetics of neat PBSu and PBSu/biochar composites filled by various filler content as calculated by (**a**) Friedman method and (**b**) Vyazovkin analysis.

In this method, the value that minimizes Φ(Eα) (as defined in Equation (7)) is used to calculate E_α_. The time and temperature at which specific α values are selected (t_α,i_ and T_α,i_) are determined through precise interpolation using a Lagrangian algorithm for each temperature program. The activation energies of the crystallization process are calculated using both the differential isoconversional method by Friedman and the integral isoconversional method by Vyazovkin. These methods allow for the analysis of the crystallization process, providing insights into the factors that influence crystallization and the relationship between crystallization temperature and time [37,38,40,41,42]. Figure 7 shows the *E_α_* values of neat PBSu and PBSu/biochar composites filled with various filler contents versus the degree of conversion *α* by using the above-mentioned isoconversional methods. It was found that the activation energy increases with increasing the degree of conversion. This suggests that the crystallization process becomes more difficult as it proceeds, indicating that the polymer system has a complex crystallization mechanism. Using Vyazovkin’s integral isoconversional method, the activation energy for the PBSu/biochar composites in Figure 7b was found to follow a trend similar to that of the activation energy found using Friedman’s differential isoconversional method (shown in Figure 7a). This implies that the two approaches yield comparable results for the activation energy of crystallization in these materials. In detail, the E_α_ values of neat PBSu and PBSu/biochar composites are negative and increase with increasing degree of conversion; this behavior is attributed to the nucleation control during the process. Nucleation is the initial step in this process, in which crystalline structures begin to form within the polymer matrix. It is a crucial step in the crystallization process because it determines the number and size of the crystalline domains that will form. Crystalline domains are regions within a material that are composed of regularly arranged, organized molecules. The number and size of these domains can significantly impact the properties of the material, such as its strength, stiffness, and transparency. When nucleation is the rate-determining step, the activation energy is expected to be negative and to increase with increasing degree of conversion. This is because the nucleation rate is sensitive to undercooling (the difference between the temperature of the polymer and its crystallization temperature), and the activation energy for nucleation is typically lower than that for growth. The undercooling diminishes as the degree of conversion rises, which in turn causes the nucleation rate to drop and the activation energy to rise. The PBSu/1% biochar and PBSu/2.5% biochar outside their early stages of crystallinity present lower activation energy values than the neat PBSu, suggesting that the addition of 1 and 2.5 wt.% of the filler accelerates the crystallization process. Wang et al. [43] showed that the heterogeneous nucleation of silicon nitride particles increased the crystallization rate of PBSu; the strong ability of silicon nitride particles to form nuclei lowered the activation energy of PBSu crystallization by a large amount and increased the efficiency of crystallization. Nucleation and crystal growth are the two processes that govern the crystallization process. The biochar particles in the matrix act as nucleation agents to help the early stages of crystallization happen. However, the PBSu/5% biochar composite presents higher values of activation energy compared to those of neat PBSu and PBSu/biochar composites. With a higher filler content of biochar in the PBSu matrix, there are more nucleation sites, but the mobility and diffusion of the PBSu chains are reduced, limiting the growth of the PBSu crystallites. It is hypothesized that these two mechanisms counteract each other at concentrations. Thus, the sufficient percentage of filler, 5 wt.% biochar, drastically affects the crystallization rate. The study by Filizgok et al. [44] examined the impact of nanofillers on the crystallization behavior of PBSu polymer, including carbon nanotubes, carbon black, and fullerene. It was found that while all types of fillers can act as sites for heterogeneous nucleation, they also have a physical limiting effect. This effect is likely due to the interaction and obstruction of the fillers with PBS molecules, which hinders the movement of the molecules towards the crystal surface. Gao et al. [17] investigated the influence of magnesium hydroxide sulfate whisker on the crystallization of PBSu and found that the presence of the whisker may hinder the movement of PBSu polymer chains during crystallization, causing an increase in the activation energy. This suggests that the development of crystals is more likely to occur in the presence of whisker particles in the early stages of crystallization. As the amount of crystallization increases, the process becomes more difficult.

#### 3.4.2. Model Fitting Methods of Avrami–Erofeev Equation, Nakamura Crystallization Model, and Šesták–Berggren Model for PBSu/Biochar Composites

Kinetic analysis is a powerful tool for understanding the mechanisms and factors that control the reaction rate of a process that is initiated by a change in temperature. It is an important consideration in many fields, including material science, where the reaction rate can have a significant impact on the final properties of a material. The reaction rate in kinetic analysis is parametrized by two main variables: the degree of conversion, which represents the fraction of the total conversion in a physical property during a process, and the temperature, T:

(9) $$\frac{\mathrm{da}}{\mathrm{dt}}=k\left(T\right)f\left(\alpha \right)$$

where k(T) is the reaction rate constant; it describes the dependence of the process rate on temperature. f(α) characterizes the dependence of the process on the degree of conversion. This equation helps to explain how the reaction rate of a process is influenced by temperature and the degree of conversion. By knowing the values of k(T) and f(α), it is possible to predict the reaction rate of the process and the resulting properties of the material. Equation (9) is applied for a single-reaction mechanism process. The overall transformation process can involve multiple mechanisms characterized by different k(T) and f(α). To accurately figure out the rate of a reaction, the rate constant and the reaction model must be given. The rate constant, or the rate at which a chemical or physical process happens, is frequently described using the Arrhenius equation. The equation takes the form:

(10) $$k\left(T\right)=A\cdot e^{-E/\mathrm{RT}}$$

where E is the apparent activation energy (kJ/mol), R the gas constant (8.314 J/mol·K), A the pre-exponential factor (s^−1^), and T the absolute temperature (K). For the kinetic analysis of non-isothermal experiments with a constant heating rate, Equation (9) can be modified as follows:

(11) $$\frac{\mathrm{da}}{\mathrm{dt}}=\frac{A}{\beta }\cdot e^{\frac{-E}{\mathrm{RT}}}f\left(\alpha \right)$$

where β represents the applied heating/cooling rate.

It is common practice to employ the Avrami equation to explain how polymers crystallize under both isothermal and non-isothermal experimental conditions. It was developed by Avrami, who proposed a theory on phase transformation kinetics based on the assumption that the new phase is nucleated by the already existing nucleation sites in the previous phase. Equation (12) describes the degree of conversion:

(12) $$\alpha \left(t\right)=1-{\;e}^{-k\left(T\right)\cdot t^{n}}$$

The differential form of the JMAEK model is given by the equation:

(13) $$\frac{d\alpha }{\mathrm{dt}}=n\cdot k\left(T\right)\cdot \left(1-\alpha \right)\cdot {\left[-\ln\left(1-\alpha \right)\right]}^{\frac{\left(n-1\right)}{n}}$$

The Avrami equation in a double logarithmic form allows for the analysis of the nucleation and growth processes at a fixed crystallization temperature. It can provide insights into the factors that influence the crystallization process and the relationship between crystallization temperature and time [45,46]:

(14) $$\log\left[-\ln\left(1-\alpha \right)\right]=\mathrm{logk}\left(Τ\right)+\mathrm{nlogt}$$

where α is the relative degree of crystallinity; t is the time from the start of phase transformation, k(Τ) is the crystallization rate constant, n is the Avrami exponent, which depends on the nucleation process and the shape of the crystalline entities being grown. The rates of spherulite growth and nuclei formation, which are crucial processes in the crystallization process, are affected by these variables. The main assumptions of this model are that the phase change occurs by nucleation and growth, with isotropic growth and random nucleation, respectively. The JMAEK equation modified by Jeziorny is the most widely used method in the literature to study the non-isothermal melt and/or cold polymers’ crystallization. Considering the non-isothermal crystallization of the process, Jeziorny modified the JMAEK method as follows:

(15) $$\mathrm{LogK}\left(T\right)=\frac{\log\;k\left(T\right)}{\beta }$$

where β is the cooling rate and K(T) is the kinetic crystallization rate constant.

The linear form of the JMAEK equation is frequently used in published crystallization studies to calculate the values of kinetic parameters because it is simple and does not require any initial assumptions. However, this fitting method can lead to inaccuracies in the resulting parameters. The models mentioned previously are designed to describe processes that occur through a single mechanism. However, when using modified versions of the JMAEK equation, it can be problematic to assume a multi-reaction mechanism rate equation, as the choice of the number of mechanisms and the reaction model can make the process more complex. Many reactions and phase transformations involve multiple mechanisms, each with its own set of kinetic parameters or even different reaction models [47]. In addition, the parameters derived from these models are specific to each heating/cooling rate. Thus, the Avrami theory presents several limitations, suggesting that the modified JMAEK equation may not be suitable for accurately describing the crystallization kinetics of PBSu under non-isothermal conditions. This is because the modified JMAEK equation goes against the fundamental idea of equating physical quantities, as pointed out by Vyazovkin.

Therefore, multivariate nonlinear regression methods have been developed to study the kinetics of both single-mechanism and multi-mechanism processes. Multivariate nonlinear regression methods are statistical techniques that are used to analyze the kinetics of processes that involve multiple variables and mechanisms. These methods are particularly useful for studying systems that exhibit complex behavior, such as single-mechanism and multi-mechanism processes. Single-mechanism processes involve a single mechanism of reaction, such as nucleation and growth, layer growth, grain growth, or volume growth. These processes can be described using the Avrami–Erofeev equation or other mathematical models. Multi-mechanism processes involve multiple mechanisms of reactions that occur simultaneously or sequentially. These processes are often more complex and may require more advanced methods of analysis. Multivariate nonlinear regression methods can be used to extract information about the different mechanisms and how they interact with each other. These methods allow for the simultaneous fitting of models to experimental data with different heating/cooling rates, resulting in a single set of kinetic parameters for each mechanism. Additionally, there are no limitations on the combination of different models or the complexity of each model. These techniques are useful for analyzing complex reaction pathways in different processes, such as crystallization, and are usually more accurate than those that just optimize a single parameter.

**Figure 8:**
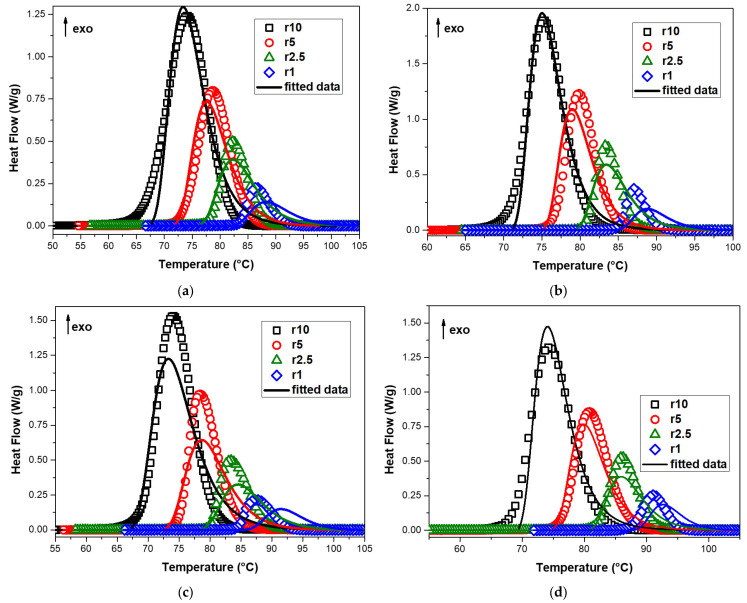
Heat flow curves of (**a**) neat PBSu, (**b**) PBSu/1% biochar, (**c**) PBSu/2.5% biochar, and (**d**) PBSu/5% biochar composites versus temperature and the corresponding fitting of multivariate nonlinear regression of the JMAEK model for single mechanism.

In this work, the n-dimensional nucleation model of the Avrami–Erofeev equation (An) was used to study the non-isothermal crystallization kinetics of neat PBSu and PBSu/biochar composites. This method employs a sixth degree Runge–Kutta approach in a modified Marquardt procedure to solve a system of differential equations relevant to the reaction types and/or their combinations. It can be used on multiple heating rate measurements at the same time to figure out the kinetic triplet of a single or multiple reaction mechanisms. Figure 8 shows the heat flow curves of neat PBSu, PBSu/1% biochar, PBSu/2.5% biochar, and PBSu/5% biochar composites versus temperature and the corresponding fitting of multivariate nonlinear regression of the JMAEK model for a single mechanism using all the cooling rates simultaneously. It was observed that the resulting fitting of the mathematical model to the data was poor, with low R2 values of 0.97314, 0.97071, 0.94921, and 0.97311 for neat PBSu, PBSu/1% biochar, PBSu/2.5% biochar, and PBSu/5% biochar composites, respectively. This indicates that the mathematical model being used does not accurately describe the data for these materials. The Avrami technique can shed light on a number of fundamental crystallization mechanisms, but it is unable to adequately describe non-isothermal crystallization behavior due to temperature fluctuations. The Avrami equation was made for primary crystallization, and it does not take into account the process of diffusion.

The Nakamura crystallization model is based on the Avrami–Erofeev nucleation model and Hoffman–Lauritzen crystal growth theory. A mathematical model called the Nakamura crystallization model is utilized to explain the crystallization kinetics of various materials [18,48,49,50,51]. The Avrami equation has been expanded by Nakamura et al. [52] to describe the transformation process that takes place in non-isothermal crystallization based on isokinetic conditions. While the nucleation rate and the growth rate both depend on time, the number of activated nuclei is thought to be independent of temperature. If the reaction constant K(T) at the temperature T is known, then the Nakamura equation for the degree of crystallinity α can be easily obtained using Equations (9) and (13) for the cooling rate β:

(16) $$\alpha \left(Τ\right)=1-\exp\left[-{\left(\frac{1}{\beta }\int_{Τ\left(0\right)}^{Τ\left(t\right)}K\left(T\right)dΤ\right)}^{n}\right]$$

For the analytical dependence of K(T), the Hoffman–Lauritzen (HL) theory can be used. The Hoffman–Lauritzen theory is commonly applied to study the crystallization process of polymers due to its simple analytical form that connects microscopic parameters with macroscopic observations. The “chain folding” theory is the basis for a model that shows how the growth rate of a crystal changes as a function of its temperature. The growth model says that once the nucleus, which acts as a growth surface (primary nucleation), is made, the crystal starts to grow in the same direction as the surface. At the same time, the existing crystals make new nuclei, which add to the length of the polymer chain (this is called “secondary nucleation”). The crystal growth parameters can be studied using non-isothermal DSC crystallization data using the following equation:

(17) $$K\left(T\right)=A\cdot \exp\;\left[-\left(\frac{U^{*}}{R\cdot \left(T-T_{\infty }\right)}\right)\right]\cdot \exp\left[\frac{-\mathrm{Kg}}{T\cdot \left(\Delta T\right)\cdot f}\right]$$

In this model, the constant preexponential factor A, the transport activation energy U*, and the kinetic parameter for nucleation Kg are all variables that affect the linear growth rate of the crystal. The transport activation energy U* represents the energy required for a segment to move to the growing front of the crystal, and has a universal value of 6.3 kJ/mol. The hypothetical temperature T indicates the temperature at which viscous flow stops, which is typically assumed to be 30 K below the glass transition temperature T_g_. The model also takes into account the chosen crystallization temperature T_c_ and the universal gas constant R = 8.314 J/(Kmol). ∆T = $T_{m}$ − T is the undercooling from melting point $T_{m}$ and f = 2T/(T_m_ + T) represents correction factor. In this study, the non-isothermal crystallization kinetics was analyzed using the Nakamura method in combination with the Hoffman–Lauritzen theory. This was performed using Equations (16) and (17):

(18) $$\frac{d\alpha }{\mathrm{dt}}=n*\left(1-\alpha \right)*\left[{\left[-\ln\left(1-\alpha \right)\right]}^{\frac{n-1}{n}}\right]*A*\exp\left(\frac{-U}{R\left(T-T_{\infty }\right)}\right)*\exp\left(\frac{K_{g}}{T\cdot \mathrm{Tf}}\right)$$

**Figure 9:**
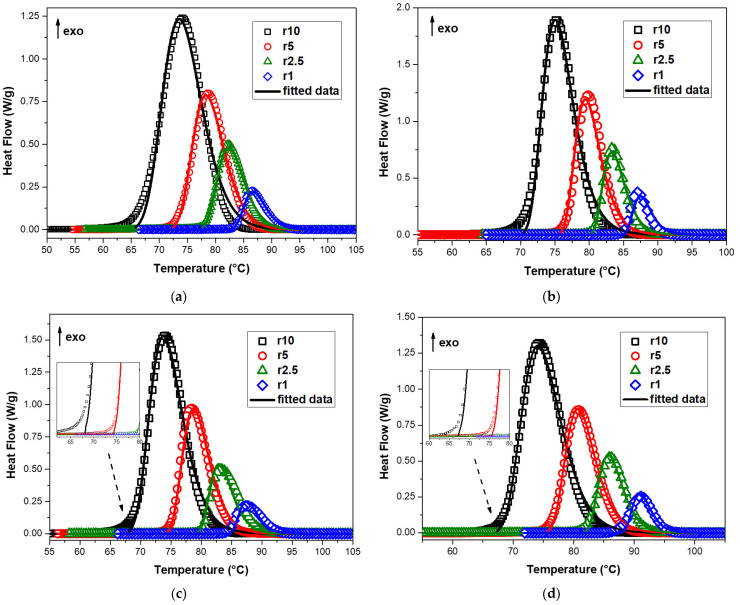
Heat flow curves of (**a**) neat PBSu, (**b**) PBSu/1% biochar, (**c**) PBSu/2.5% biochar, and (**d**) PBSu/5% biochar composites versus temperature and the corresponding fitting of multivariate nonlinear regression of the Nakamura (Nk) model with Hoffman–Lauritzen theory.

The comparison of the experimental and the corresponding fitting of multivariate nonlinear regression of the Nakamura (Nk) model with Hoffman–Lauritzen theory is presented in Figure 9. In this study, the Nakamura/Hoffman–Lauritzen model’s parameters for neat PBSu and PBSu/biochar composites were determined using the nonlinear regression method. Fast Scanning Calorimetry was used by Lapuk et al. [49] to examine the glassy amorphous forms of the drugs dopamine hydrochloride and atenolol. The measured and predicted data match up best with the Nakamura crystallization model, which is based on the Avrami equation for nucleation and the Hoffman–Lauritzen crystallization theory. Additionally, Seo et al. [48] used the dual Nakamura model for primary and secondary crystallization applied to non-isothermal crystallization of poly(ether ether ketone). Table 4 contains a list of the parameters that were obtained. Figure 9 illustrates how well the predictions of the crystallization process for each of the PBSu/biochar composites under investigation match the experimental facts. The experimental results and the predictions of the theoretical model diverge near the end of the crystallization process. The model tends to underestimate the crystallization rate, which may be caused by secondary crystallization that is not considered in the Nakamura model. This effect has been observed in previous studies and is thought to be related to the limitations of the model in describing certain types of crystallization processes [53]. According to Table 4, the K_g_ values of the PBSu/1% biochar and PBSu/2.5% biochar composites are higher than that of neat PBSu. This means that these composites have higher barriers for secondary nucleation. Additionally, the energy barrier for crystal formation was higher in neat PBSu compared to that of the PBSu/5% biochar composite. The half crystallization time, t_1/2_, and the measured crystallization rates from the isoconversional studies do not line up with the suggested accelerating impact. It is evident that the Nakamura (Nk) model coupled with the Hoffman–Lauritzen theory cannot adequately fit the observed dependency.

The Hoffman–Lauritzen theory for diffusion and the Šesták–Berggren model for the nucleation term combine two different theories to account for the different mechanisms involved in the crystallization process. This model has become a widely used tool in the field of material science for understanding and optimizing the crystallization process in a range of polymer systems. Guigo et al. [53] used several models, such as the Šesták–Berggren, Avrami and Ozawa models, to explain the degree of crystallization dependence of poly(ethylene 2,5- furandicarboxylate). It was shown that the model-free method might be able to explain new crystallization phenomena that the traditional Hoffman–Lauritzen theory or Avrami equations do not cover. Then, a new equation has been put forward to model other processes that happen at the end of crystallization, such as the combination of the Hoffman–Lauritzen theory for diffusion and the Šesták–Berggren model for the nucleation process. It is a mathematical formula that is frequently used to understand the way in which polymers crystallize when the temperature is not constant. It has been found to be particularly effective in accurately predicting the crystallization behavior of many polymers, especially when the Nakamura model tends to underestimate the crystal fraction near the end of crystallization [53]. The Šesták–Berggren model [54], which is an autocatalytic type of transformation, includes an additional parameter that indicates the growth of crystals as new crystalline nuclei form and represents a variety of reaction models. The Šesták–Berggren model is a widely used model for the analysis of thermal data obtained from DSC experiments. This model is based on the Avrami equation, which describes the kinetics of crystallization in terms of the nucleation rate and the growth rate of the crystals. The parameter model equation is described in Equation (19):

(19) $$f\left(\alpha \right)=\alpha^{m}\cdot {\left(1-\alpha \right)}^{n}\cdot {\left[-\ln\left(1-\alpha \right)\right]}^{p}$$

which depends on the combination of m, n, and p, representing a number of different reaction models. Τhe parameters m and n are defined as the relative contributions of the acceleratory and decay regions of the kinetic process.

It is normally used in the truncated form (p = 0):

(20) $$f\left(\alpha \right)=\alpha^{m}\cdot {\left(1-\alpha \right)}^{n}$$

where m is the order of autocatalytic reaction and n is the order of reaction. For the Šesták–Berggren (m, n) model, the kinetic parameter ratio is calculated as p = m/n [55,56].

**Figure 10:**
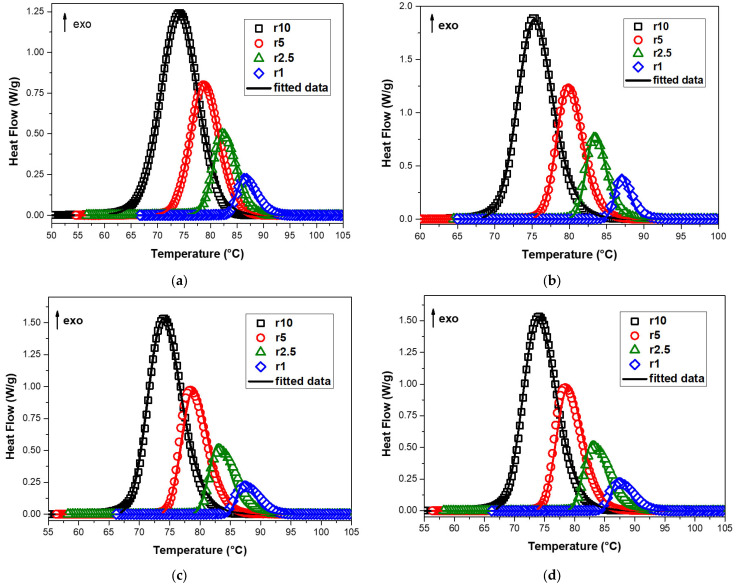
Heat flow curves of (**a**) neat PBSu, (**b**) PBSu/1% biochar, (**c**) PBSu/2.5% biochar, and (**d**) PBSu/5% biochar composites versus temperature and the corresponding fitting of multivariate nonlinear regression of the Šesták–Berggren model with Hoffman–Lauritzen theory.

In this work, the non-isothermal crystallization kinetics of neat PBSu and PBSu/biochar composites was studied by using the Hoffman–Lauritzen theory and the Šesták–Berggren model for diffusion and nucleation, respectively. Figure 10 shows the heat flow curves of neat PBSu, PBSu/1% biochar, PBSu/2.5% biochar, and PBSu/5% biochar composites versus temperature and the corresponding fitting of multivariate nonlinear regression of the Šesták–Berggren model with Hoffman–Lauritzen theory for a single mechanism using all the cooling rates simultaneously. Figure 10 illustrates the good agreement between the predicted values for the crystallization of neat PBSu and PBSu/biochar composites made using the Hoffman–Lauritzen theory and the Šesták–Berggren model for the nucleation. In particular, at the end of the crystallization process, where the simulated curves closely resemble the experimental curves, the combination of the aforementioned models accurately captures the crystallization behavior of PBSu and PBSu/biochar composites. This supports the hypothesis that additional crystallization processes that take place at higher degrees of conversion can be correctly accounted for by the Šesták–Berggren model with Hoffman–Lauritzen theory. These results are consistent with those obtained using the iso-conversional methods, which suggest that the melt crystallization mechanism is complex and involves multiple steps with different contributions and activation energies. These models take into account the different variables that affect crystallization, including how the process is affected by temperature.

According to the results in Table 5, the addition of biochar significantly affects the PBSu matrix’s crystallization behavior. This is demonstrated by the changes in the reaction order parameters m and n, which describe the rate of the crystallization process. The K_g_ parameter of neat PBSu was found to be 0.7099 K^2^, very close to the values presented in the literature, 0.8431 K^2^ by Hwang et al. [57] and 0.8031 K^2^ by Soccio et al. [58]. The PBSu/1% biochar composite was found to have a lower nucleation parameter K_g_ than neat PBSu, PBSu/2.5% biochar, and PBSu/5% biochar composites. This indicates that heterogenous nucleation phenomena occur during the crystallization of the PbS/1% biochar composite. The values of Kg obtained from the Šesták–Berggren model indicate that a lower amount of biochar in the PBSu matrix can facilitate the nucleation of polymer chains and the initiation of crystallization at higher temperatures during melt crystallization. The formation of critically sized nuclei in this composite likely requires less energy. However, the K_g_ parameter values increase with increasing filler content. The addition of 5 wt.% biochar could change the secondary nucleation constant. It could also slow down the movement of molecular chains. Additionally, the pre-exponential factor A of the PBSu/1% biochar composite presents a lower value than that of neat PBSu following the calculated E values. This observation is particularly intriguing because it suggests that the addition of biochar to the PBSu polymer matrix can have a significant impact on the crystallization behavior of the composite. The material may become more appropriate for particular applications by virtue of the acceleration of the crystallization process, which can have significant effects on the material’s mechanical and thermal properties. This means that the rate constant of the PBSu/1% biochar composite is significantly larger than those of neat PBSu, PBSu/2.5% biochar, and PBSu/5% biochar, accelerating the crystallization process. This suggests that as biochar content is increased, the composite’s rate constant lowers, slowing the crystallization process.

The parameter m is the autocatalytic order, n is the reaction order, and p the kinetic ratio parameter, calculated by the fitting of the data with the Šesták–Berggren model/Hoffman–Lauritzen model. On the one hand, the parameter p was found to be 0.60, 0.82, 0.71, and 0.74 for neat PBSu, PBSu/1% biochar, PBSu/2.5% biochar, and PBSu/5% biochar, respectively. This indicates that the rate constant of the PBSu/1% biochar composite is significantly larger than those of neat PBSu, PBSu/2.5% biochar, and PBSu/5% biochar, accelerating the crystallization process. On the other hand, the kinetic parameter ratio as calculated by using the equation p = m/n was found to be 0.62, 0.82, 0.74 and 0.78 for the different composites. These values were very close to those calculated from the simulation, indicating the effectiveness of this model in accurately predicting the crystallization behavior of the PBSu/biochar composites. The parameter p is connected with Avrami nucleation dimension n; it was found to be 2.15, 2.84, 2.53, 2.65 for neat PBSu, PBSu/1% biochar, PBSu/2.5% biochar and PBSu/5% biochar composites, respectively, in accordance with the literature [59]. This indicates that the PBSu/biochar composites have a higher nucleation density than neat PBSu, which can be attributed to the presence of biochar particles acting as nucleation sites. Lee et al. [60] analyzed the isothermal bulk crystallization kinetics of neat PBSu by using the Avrami analysis; a value of 2.2 was reported for PBSu. According to the analysis, the probable nucleation and growth mechanisms for neat PBSu and PBSu/biochar composites are site saturation in 2D and 3D, respectively. It is well known that the value of the parameter n in the Avrami equation is influenced by both the mechanism of nucleation and the morphology of crystal growth. An n value of 2 means that the crystal growth is sporadic and spherical, and occurs from nuclei. The increase in n values for PBSu/biochar composites suggests that the crystallization mechanism changes due to the heterogeneous nucleation of PBSu caused by the presence of biochar. The biochar filler appears to act as a heterogeneous nucleation agent for PBSu, which is supported by the higher nucleation activity observed in the PBSu/biochar composites. This indicates that the crystallization process is more complex in these composites compared to neat PBSu. However, the PBSu/1% biochar increases more the Avrami exponent n compared to those of PBSu/2.5% and PBSu/5% biochar composites. This fits with the idea of an accelerating effect, as well as the observed crystallization rates found through isoconversional analyses and the time it takes for half of the crystals to form. The filler probably changed the shape of the crystals, which is why the n values of PBSu/2.5% biochar and PBSu/5% biochar composites went down. The presence and increase in biochar content in composite materials would not allow these entities to easily develop in all directions. Once again, the 5% wt. played a dual role, acting as both a nucleating agent to promote nucleation and a physical hindrance to retard chain segment transport during non-isothermal crystallization. Similar behavior has been reported in the literature [38].

It was found that the glass transition temperature of neat PBSu is 35 °C. This value agrees well with research results [19,58,61]. Glass transition temperatures of the PBSu/biochar composites slightly decrease with increasing biochar content, possibly due to the increase in free polymer volume caused by the concentration of polymer chains around the biochar particles. This concentration of polymer chains around the biochar particles may also suggest a partial immobilization of the polymer chains, leading to a decrease in T_g_. Additionally, the melting temperature of neat PBSu was found to be 114.91 °C, very close to the value calculated earlier (Table 1). The addition of biochar filler to PBSu results in a decrease in the melting temperature of the composites. Once again, the addition of 5 wt.% of biochar filler shifts the T_m_ to considerably lower temperatures compared to those of neat PBSu and PBSu/biochar composites filled with 1 wt.% and 2.5 wt.%. The concentration of polymer chains around the biochar particles is likely the reason for the increase in free polymer volume and partial immobilization of the chains.

The use of the Šesták–Berggren/Hoffman–Lauritzen model to study non-isothermal crystallization behavior in PBSu/biochar composites is a novel approach that has not been previously explored. This model provides a detailed description of the kinetics of crystallization, including the parameters m, n, and p, which can be used to predict the behavior of the composite material under various conditions. By examining the Šesták–Berggren/Hoffman–Lauritzen model in the context of PBSu/biochar composites, we can gain insight into the factors that affect the crystallization behavior of these materials. This information can be used to optimize the processing conditions for PBSu/biochar composites, as well as to develop new composite materials with tailored properties.

## 4. Conclusions

This study looked at the non-isothermal crystallization kinetics of neat PBSu and PBSu/biochar composites by using isoconversional and model-based methods. The results of this study show that the addition of biochar to PBSu affects the crystallization behavior of the composites. The incorporation of biochar results in a lower melting temperature and an increase in the crystallization temperature of PBSu, but it also increases the number of heterogeneous nucleation sites, leading to a higher crystallization rate. The outcomes also showed that the PBSu/biochar composites’ non-isothermal crystallization behavior could not be adequately described by the Avrami model. For the first time, two distinct models of non-isothermal crystallization for PBSu/biochar composites were examined. The non-isothermal crystallization kinetics of PBSu/biochar composites can be fitted using the Nakamura/Hoffman–Lauritzen model as well as the Šesták–Berggren/Hoffman–Lauritzen model. However, the Nakamura model underestimates the crystal fraction near the end of crystallization because it ignores slower secondary crystallization kinetics. Thus, the Šesták–Berggren/Hoffman–Lauritzen model can predict the kinetics of crystallization for different rates of cooling under non-isothermal conditions. In detail, the isoconversional methods and the multivariate nonlinear regression analysis using the Šesták–Berggren models with Hoffman–Lauritzen theory suggest that the crystallization mechanism of PBSu/biochar composites is complex and involves both nucleation and growth processes. According to the model-based analysis, the biochar filler appears to have raised the energy barrier for crystal formation, leading to a slower crystallization rate at higher filler contents. The addition of biochar changes both the shape of the crystalline structures and the kinetic parameters. Τhe calculated K_g_ parameter of neat PBSu was found to be 0.7099 K^2^, very close to the values presented in the literature. The glass transition temperature and the melting temperature of neat PBSu were found to be 35 °C and 114.91 °C, respectively, very close to the values measured. The values of Kg obtained from the Šesták–Berggren model indicate that a lower amount of biochar in the PBSu matrix can facilitate the nucleation of polymer chains. Additionally, the pre-exponential factor A of the PBSu/1% biochar composite presents a lower value than that of neat PBSu following the calculated activation energy values. The Avrami nucleation dimension n of PBSu/5% biochar composite was found to be 2.65, indicating that the crystallization process is complex in the composites. The m/n ratio goes up, and the Avrami exponent goes down. The 5% wt. played a dual role, acting as both a nucleating agent to promote nucleation and a physical hindrance to retard chain segment transport during non-isothermal crystallization. Overall, the incorporation of biochar filler had a complex effect on the crystallization behavior of PBSu, which highlights the importance of using multiple methods to fully understand the underlying mechanisms. This study contributes to a deeper understanding of non-isothermal crystallization and presents an overall method for modeling crystallization of PBSu biochar composites under process conditions in order to optimize their production and improve their thermal and mechanical properties.

## Figures and Tables

**Table 1:** Melting temperature, T_m_, enthalpy of fusion, ΔH_m_, and absolute degree of crystallinity, X_c_, of neat PBSu and PBSu/biochar composites filled with various filler content at a heating rate of 20 °C/min.

A/A	T_cc_ (°C)	T_m_ (°C)	ΔH_m_ (J/g)	X_c_ (%)
Neat PBSu	98.8	115.2	70.1	33.4
PBSu/biochar 1 wt.%	98.4	114.9	73.2	35.2
PBSu/biochar 2.5 wt.%	98.9	114.8	75.9	37.1
PBSu/biochar 5 wt.%	96.0	113.5	76.0	38.1

**Table 2:** The crystallization temperature, T_c_, and the crystallization enthalpy, ∆H_c_, of PBSu/biochar composites under non-isothermal crystallization.

Cooling Rate (°C/min)	PBSu		PBSu/Biochar 1 wt%		PBSu/Biochar 2.5 wt%		PBSu/Biochar 5 wt%	
	T_c_ (°C)	ΔH_c_ (J/g)	T_c_ (°C)	ΔH_c_ (J/g)	T_c_ (°C)	ΔH_c_ (J/g)	T_c_ (°C)	ΔH_c_ (J/g)
10	74.1	71.4	75.2	71.7	74.0	69.0	74.3	69.6
5	78.7	70.5	79.8	71.1	78.4	69.4	80.8	72.4
2.5	82.3	72.5	83.4	69.9	83.3	70.9	86.1	71.6
1	86.5	69.2	87.1	69.9	87.4	69.1	91.1	70.3

**Table 3:** Half time of crystallization, t_1/2_, for neat PBSu and PBSu/biochar composites filled with various filler content.

Cooling Rate (°C/min)	Half Time of Crystallization, t_1/2_ (min)			
	PBSu	PBSu/Biochar 1 wt.%	PBSu/Biochar 2.5 wt.%	PBSu/Biochar 5 wt.%
10	33.6	33.4	33.6	33.5
5	63.2	63.0	63.2	62.7
2.5	121.9	121.5	121.4	120.4
1	295.9	295.7	293.2	291.8

**Table 4:** The Nakamura/Hoffman–Lauritzen model parameters of neat PBSu and PBSu/biochar composite.

Sample	K_g_ (10^5^ K^2^)	Log(A) s^−1^	Dimension, n	T_m_ (°C)	T_g_ (°C)	R^2^
Neat PBSu	1.6234	4.31	2.40	129.62	−35	0.99468
PBSu/biochar 1 wt.%	1.9195	4.84	3.42	132.82	−35	0.99382
PBSu/biochar 2.5 wt.%	1.8199	4.47	3.26	133.5	−35	0.99514
PBSu/biochar 5 wt.%	1.3347	3.34	3.67	133.5	−35	0.99532

**Table 5:** Parameters of the Šesták–Berggren model with Hoffman–Lauritzen theory for the crystallization curves from the melt of neat PBSu and PBSu/biochar composites.

Sample	PBSu	PBSu/Biochar 1 wt.%	PBSu/Biochar 2.5 wt.%	PBSu/Biochar 5 wt.%
Kg (10^5^ Κ^2^)	0.7099	0.4337	1.1984	1.3808
Log(A) s^−1^	3.32	2.92	4.18	4.06
n	1.02	0.90	0.89	0.91
m	0.63	0.73	0.66	0.71
p	0.60	0.75	0.71	0.74
T_m_	114.91	114.72	114.71	114.06
T_g_	−35.0	−34.8	−34.5	−33.9
R^2^	0.99906	0.99901	0.99529	0.99897

## Data Availability

All the data of this study are included in the manuscript.
